# Biological markers of impending psychosis :A systemaic literature review

**DOI:** 10.1192/j.eurpsy.2023.563

**Published:** 2023-07-19

**Authors:** M. Stambouli, B. N. Saguem, J. Nakhli

**Affiliations:** psychiatry department, Farhat hached hospital, sousse, Tunisia

## Abstract

**Introduction:**

Despite the encouragingresearch findings concerning the predictive validity of the psychosis risk criteria, they are still insuffisant to justify treatment recommandations and attempts at preventive interventions for this early, non-specific illness phase. So far, diagnosis of the prodromal symptoms is largely based on patient reports of symptoms and/or collateral reporting, and yet remains relatively devoid of objective, brain-based biological markers. Adding specific predictors and biological markers to the clinical psychosis risk approach that could increase the predictive power of current psychosis risk criteria is a crucial step for early intervention efforts.

**Objectives:**

We reviewed all studies examining the biological markers in subjects with an ‘’At Risk Mental State” (ARMS) for psychosis and we discussed their predictive psychosis transition value.

**Methods:**

A systematic search of the literature was performed using PubMed. The key words «Early psychosis / Prodromal symptoms / At risk mental state» in combination with «Biomarkers / Inflammatory markers / Stress» were used for the search.

**Results:**

We selected 18 papers: 2 literature reviews, 7 cross-sectional studies and 9 prospective studies. Aiello et al. (2012) reviewed studies examining biological markers of the stress response in the relatives of psychotic patients and in individuals at Ultra-High Risk (UHR) for psychosis. Karanikas and Garyfallos (2015) systematically reviewed data concerning the role of cortisol in patients at risk for psychosis mental state and its associations with psychopathological correlates. There was no review available of all biomarkers.

The biological markers that have been reported are mainly stress markers, endocrine and inflammatory markers.

Serum or salivary cortisol was the most studied marker. Studies stated that UHR subjects had higher cortisol levels than controls and that there was a positive correlation between cortisol levels and the severity of symptoms. Results of prospective studies were controversial.

High levels of prolactin were reported by 3 studies, including a prospective onewhichconcluded that prolactine may be considered as a biological marker of transition.

A prospective study found higher levels of interleukin-6 (IL-6) in UHR subjects than in controls. This study also reported significantly higher IL-6 levels in subjects who transitioned to psychosis compared to those who did not.

**Conclusions:**

Despite advances in mental health research, there is still a lack of biological markers with clinical utility. The results of current studies should be interpreted with caution due to the small sample size of the prospective studies. The latter open up research perspectives in the identification of UHR subjects, taking into account other markers to better describe the profile of those who will present transition to psychosis.

**Disclosure of Interest:**

None Declared

